# A genetic variant of adenylate cyclase 7 associated with ulcerative colitis shows impaired function and G-protein-coupled receptor signaling

**DOI:** 10.1007/s00439-026-02848-z

**Published:** 2026-07-04

**Authors:** Gabriele Loers, Selen Cangüzel, Sebastian Rading, Christian Kubisch, Meliha Karsak

**Affiliations:** 1 Neuronal and Cellular Signal Transduction, Institute of Human Genetics, University Medical Center Hamburg-Eppendorf, Martinistr. 52, 20246 Hamburg, Germany; 2 Institute of Human Genetics, University Medical Center Hamburg- Eppendorf, Martinistr. 52, 20246 Hamburg, Germany

## Abstract

A missense variant of adenylate cyclase 7 (AC7), p.Asp439Glu, has been significantly associated with ulcerative colitis (UC) in genome-wide association studies. Previous work suggested that this variant is reduced in expression and exhibits impaired cyclic adenosine-3′,5′-monophosphate (cAMP) synthesis, thus skewing T-cell cytokine profiles. Here, we investigated the variant’s function measuring dynamic cAMP responses in live HEK293 cells. We show that p.Asp439Glu generates significantly reduced basal cAMP levels despite normal expression. Stimulation with sphingosine-1-phosphate (S1P) and phorbol 12-myristate 13-acetate (PMA) induced a reduced cAMP increase in cells expressing mutant AC7, indicating reduced responsiveness to G-protein-coupled receptor (GPCR) and protein kinase C (PKC) activation. Western blot analysis showed altered downstream phosphorylation of cAMP response element-binding protein (CREB) and cAMP-dependent transcription factor (ATF1) in cells expressing the variant. Higher levels of phosphorylated CREB and ATF1 were observed in cells expressing p.Asp439Glu AC7 under basal conditions while stimulation with S1P had no effect on protein phosphorylation. Our findings provide direct biochemical evidence for strongly impaired AC7 function caused by the variant p.Asp439Glu. These results may pave the way for more causally defined and genotype-specific treatment strategies in UC.

**Graphical abstract:**

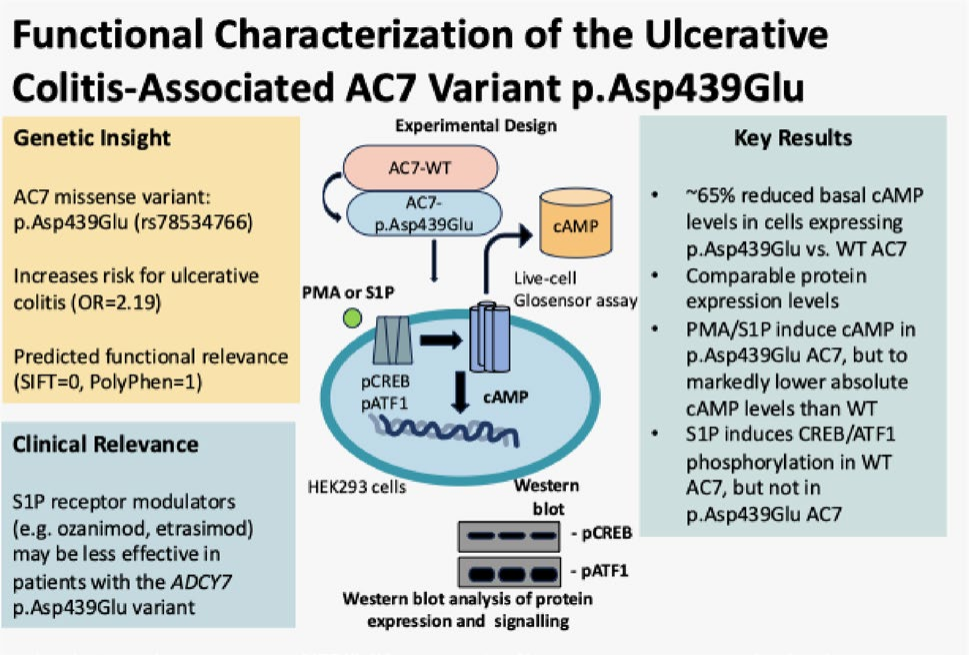

## Introduction

**Fig. 1:**
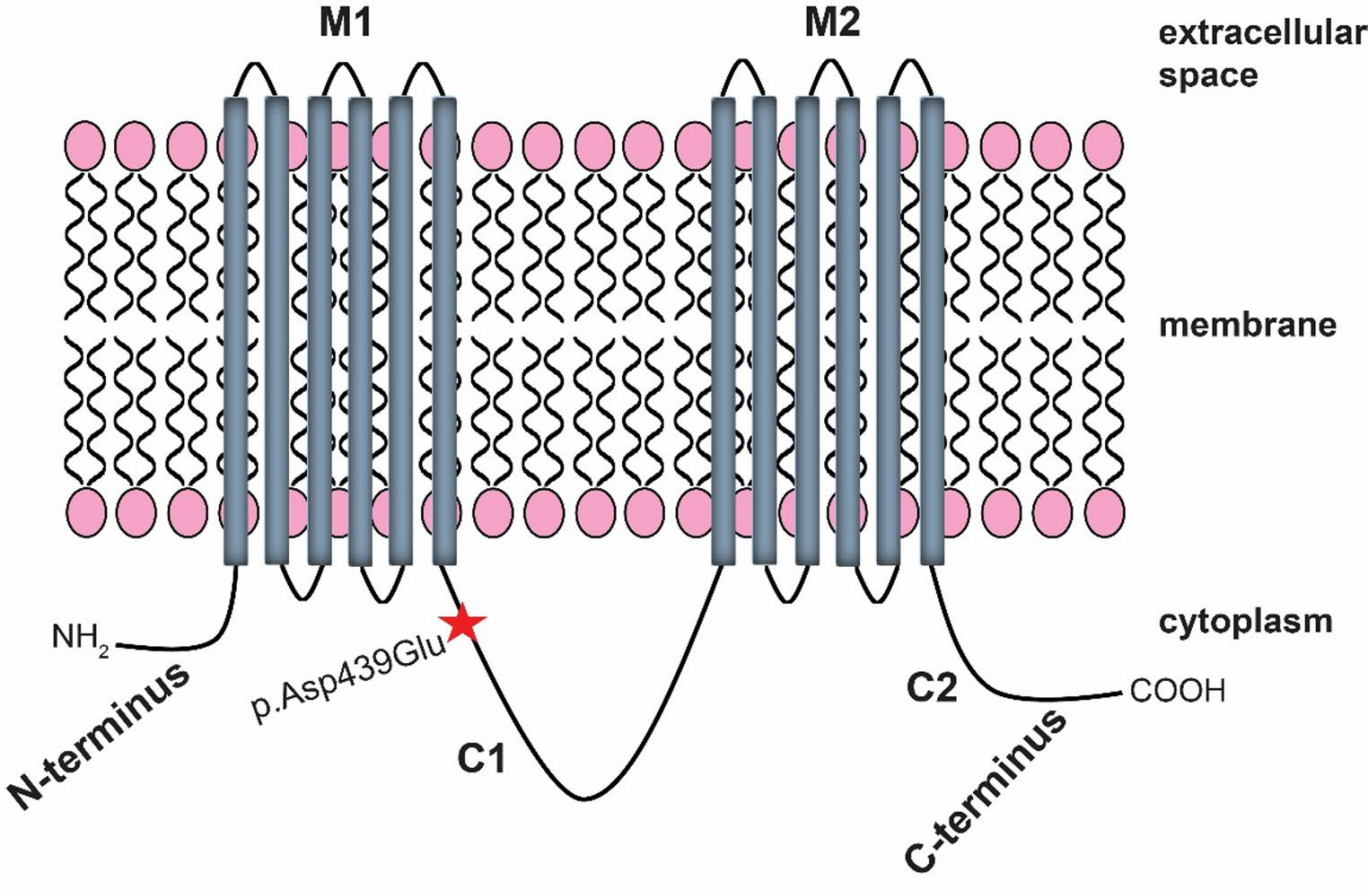
Structural organization of AC7 within the lipid bilayer. AC7 consists of a short intracellular N-terminus, two transmembrane domains (M1 and M2) each containing 6 transmembrane helixes, a large cytoplasmic domain (C1) separating the transmembrane domains, and a large C-terminus (C2). The catalytic domain is formed by subdomains of C1 and C2 and both contribute to substrate binding (Devasani and Yao 2022; Gao et al. 2024; Ostrom et al. 2022). The p.Asp439Glu exchange in the C1 region is depicted by a red star

Inflammatory bowel disease (IBD), comprising Crohn’s disease (CD) and ulcerative colitis (UC), represents a group of chronic disorders driven by complex interactions between genetic, environmental, and immune factors. Genome-wide association studies (GWAS) have identified numerous genetic loci associated with IBD susceptibility. Among these, *ADCY7*, encoding adenylate cyclase 7 (AC7), has emerged as a gene of interest. The *ADCY7* missense variant rs78534766, resulting in a p.Asp439Glu substitution in the cytoplasmic domain C_1_ (Fig. 1), is associated with a doubled risk of developing UC (odds ratio = 2.19, 95% CI = 1.75–2.74) (Luo et al. 2017). Computational analyses (SIFT, PolyPhen, and MutationTaster) suggest that this substitution impairs protein function. A reduction in AC7 function could predispose to dysregulated immune responses characteristic of UC. Recent work by Cardinale et al. demonstrated that the p.Asp439Glu variant may exhibit reduced protein expression and impaired cAMP synthesis in T-helper cells, leading to widespread transcriptomic changes including upregulation of Th2 cytokines and MHC class II molecules (Cardinale et al. 2025). These findings link AC7 malfunction to Th2 polarization, a known hallmark of UC pathology.

The membrane-bound AC7 enzyme catalyzes the conversion of adenosine-5´-triphosphate (ATP) to cAMP, a critical secondary messenger in modulating innate and adaptive immune responses, including suppression of pro-inflammatory cytokine release and regulation of immune cell trafficking (Raker et al. 2016; Serezani et al. 2008; Tavares et al. 2020). AC7 is activated in response to GPCR stimulation and after binding to G-proteins (Devasani and Yao 2022; Jiang et al. 2008; Ostrom et al. 2022). The cAMP generated by AC7 activates down-stream protein kinase A (PKA) and exchange proteins directly activated by cAMP (EPACs), leading to phosphorylation of MAP kinases and basic leucine zipper transcription factors like CREB and ATF1 and activation of the WNT/β-catenin pathway (Guo et al. 2022).

Phorbol 12-myristate 13-acetate (PMA) activates adenylate cyclases indirectly via protein kinase C (PKC), which phosphorylates the enzyme and enhances its catalytic activity (Conley et al. 2013). This PKC-dependent modulation increases the responsiveness of AC7 to Gs-coupled inputs, thereby potentiating stimulus-evoked cAMP production (Nelson et al. 2003).

GPCRs are activated by a wide range of endogenous ligands, including ions, lipids, nucleotides, amines, small molecules and peptides (Sutkeviciute and Vilardaga 2020). Due to the extensive involvement of GPCRs and their downstream signaling partners in health and disease, they are prominent drug targets for therapeutic applications. Signaling by spingosine-1-phosphate (S1P), a bioactive lipid that regulates immune and vascular systems, has become a therapeutic target in UC. S1P signals through five GPCRs, S1P receptor 1 to 5 (S1PR1–S1PR5), which mediate a range of physiological processes, including immune cell egress from lymphoid organs and vascular permeability. Agents such as ozanimod and etrasimod, which modulate S1PR1 and S1PR5, reduce lymphocyte trafficking to the gut, alleviating inflammation and promoting mucosal healing. Clinical trials have demonstrated their efficacy, with ozanimod achieving remission rates of 18.4% compared to 6% with placebo (Cardinale et al. 2025; Eden et al. 2025; Luo et al. 2017).

To date it is unknown, whether the p.Asp439Glu AC7 variant retains responsiveness to upstream signaling and if stimulation, e.g. with S1P, could be a useful therapy to treat UC patients carrying this variant. In this study, we characterized protein expression levels, the responsiveness to GPCR activation by S1P, the response to PMA stimulation and downstream cAMP signaling of the p.Asp439Glu variant. A schematic showing the structural organization of AC7 and the mutation site is shown in Fig. 1. Our findings offer insights into the molecular mechanisms linking *ADCY7* variants to UC pathogenesis and therapeutic responses.

Here, we sought to characterize the functional activity of the AC7 p.Asp439Glu variant using a real-time live-cell assay in a heterologous expression system. We focused on three key aspects: (1) basal cAMP production, (2) downstream signaling via phospho-CREB/ATF1, and (3) responsiveness to PMA and S1P stimulation.

## Materials and methods

### Plasmids and Cell Culture

Human *ADCY7* cDNA with added Flag-tag at the C-terminus was cloned by GenScript into the pcDNA3.1 vector. The p.Asp439Glu variant was introduced by site-directed mutagenesis and verified by Sanger sequencing by GenScript. HEK293 cells (ATCC) were cultured in DMEM (high glucose; ThermoFisher Scientific, Darmstadt, Germany) with 10% FBS and 1% penicillin-streptomycin (Capricorn Scientific GmbH, Ebsdorfergrund, Germany). Cells were co-transfected with either wild-type (WT) or p.Asp439Glu *ADCY7* plasmid and the Glosensor cAMP 22 F reporter (Promega, Walldorf, Germany), with the *ADCY7* plasmids alone or an empty vector using Lipofectamine 2000 (ThermoFisher Scientific).

## Live-Cell cAMP Assay

The cAMP assay was performed as described before (Zhou et al. 2023) with some modifications: Cells were seeded at a density of 1 × 10^6^ per well into 6-well plates (Sarstedt, Nümbrecht, Germany). The cells were incubated in DMEM (ThermoFisher Scientific) with 10% FCS and 1% penicillin-streptomycin overnight in a 37 °C incubator at 5% CO_2_. 24 h later, the medium was changed to DMEM with 10% FCS but without antibiotics and cells were transfected with either pcDNA3.1 or WT or p.Asp439Glu AC7 plasmid and the Glosensor cAMP 22 F reporter (Binkowski et al. 2011). 24 h after transfection, cells were transferred to poly-L-lysine coated 96-well plates (4 × 10^4^ cells per well; white 96-well microplates with µClear^®^ bottom, Greiner Bio-One, Frickenhausen, Germany) and the plates were incubated overnight in a 37 °C incubator at 5% CO_2_. Then, cells were incubated for 1 h in luciferin assay buffer consisting of Leibovitz L-15 medium supplemented with 1% FCS, 5 mM beetle luciferin, and 100 µM 3-isobutyl-1-methylxanthine (IBMX) in the dark at room temperature. After incubation, baseline luminescence signal was measured (Mithras, Berthold, Bad Wildbad, Germany) for 40 min with 5 s measurements per well for the FSK experiments and for 20 min with 1 s measurements per well for the PMA experiments. Next, cells were stimulated with 1 µM sphingosine-1-phosphate (S1P; Biomol-GmbH, Hamburg, Germany), protein kinase C activator phorbol 12-myristate 13-acetate (PMA, 50 µg/µl; Invivogen, San Diego, USA) or treated with control solution and luminescence was recorded for 32 min or 20 min. Subsequently, cells were treated with the AC7 activator forskolin (FSK, 1 µM; Sigma-Aldrich) and then with the AC7 inhibitor MDL12330A (MDL, 500 µM; Sigma-Aldrich) and luminescence was recorded for further 32 min or 20 min. S1P was dissolved by ultrasonification in PBS pH 7.5 with 0.1% fatty-acid free BSA in a stock solution of 1 mM in silanized glassed tubes. PMA was dissolved in DMSO. For the application onto the cells, aliquots of the stocks and vehicle solutions were further diluted in Leibovitz-buffer with a final DMSO solvent concentration of 0.1% and a final compound concentration of 1 µM.

## **Raw relative luminescence unit (RLU) Normalization**

RLU values obtained in cAMP assays were normalized using two complementary approaches.

Normalization to the well-specific baseline:

For each well, the baseline RLU value was defined as the measurement at the initial time points (baseline). All subsequent RLU values in that well were divided by its own baseline, yielding fold-changes relative to the starting level. This approach allows comparison of stimulus-induced responses while accounting for well-to-well variability in basal luminescence.

Normalization to the mean WT baseline:

Alternatively, RLU values were normalized using the mean baseline of WT control wells. The baseline RLU for each WT well in the single experiment were used to calculate the mean baseline across all WT wells. RLU values from all wells and time points were then divided by this mean WT baseline, enabling comparison of absolute changes relative to a standardized WT reference.

Both normalization methods were applied to the dataset to assess either fold-changes relative to individual well baselines or absolute differences relative to the WT control baseline.

## Western Blotting

Whole-cell lysates were prepared in RIPA buffer (50 mM Tris pH 7.5, 150 mM NaCl, 1% NP40 alternative, 1 mM Na_2_P_2_O_7_) with protease/phosphatase inhibitor cocktail (Roche, Basel, Switzerland) and either directly used for SDS-gel analysis or frozen and stored at -80 °C until use. Protein concentrations were determined using the BCA test (ThermoFisher Scientific). Lysates were applied onto 10, 12 or 4–20% SDS-gels (25 µg protein/lane) and after separation proteins were transferred onto 0.2 μm nitrocellulose membranes by Western blotting. Proteins on membranes were stained with LiCOR Revert™ 700 Total Protein Stain (LiCOR Bio, Bad Homburg, Germany) to control for equal loading and membranes were blocked with 4% skim milk powder in Tris buffered saline solution containing 0.05% Tween-20 (TBST). Blots were probed with antibodies against the Flag-tag to visualize Flag-tagged AC7 proteins (Sigma-Aldrich; 1:1,000) and phospho-CREB/phospho-ATF1 (Cell Signaling Technology, Leiden, The Netherlands; 1:1,000), followed by fluorescence-coupled secondary antibodies (1:20,000 in blocking solution; LiCOR Bio) and imaging using a fluorescence reader (Odysee CLx; LiCOR Bio). Bands were analyzed using Emipria Studio 3.0 software (LiCOR Bio). To control for uneven sample loading, pipetting errors, and variations in protein transfer efficiency, total protein normalization was performed for AC7, CREB and ATF1 levels in the respective lane of each Western blot.

### Immunostaining

For immunostaining, cells were seeded at a density of 2 × 10^5^ onto poly-L-lysine coated glass coverslips in 24-well plates and grown and transfected with *ADCY7* cDNA as described above. Cells transfected with the empty vector served as control. 48 h after transfection, cells were washed once with Hank´s balanced salt solution (HBSS) and then fixed with 4% formaldehyde (w/v) in phosphate buffered saline solution (PBS) for 30 min at room temperature. After three washes in PBS, cells were blocked and permeabilized with 2% bovine serum albumin, 0.5% Triton X-100 in PBS for 1 h. To visualize Flag-tagged AC7 WT and p.Asp439Glu protein, an antibody against the Flag-tag (1:150; rabbit; Sigma-Aldrich) was applied overnight at 4 °C. After washing with PBS and cells were incubated with Oregon green 488 coupled wheat germ agglutinin (WGA; 1:200; ThermoFisher Scientific) to stain N-acetylglucosamine and sialic acid residues at the plasma membrane and Cy3-coupled anti-rabbit antibody (1:200; Jackson ImmunoResearch, Ely, UK) for 1 h at room temperature. After washing coverslips were mounted in anti-fading mounting solution containing DAPI to visualize the nuclei (RotiMount^®^ with DAPI; Carl Roth, Karlsruhe, Germany). Cells were imaged using a confocal laser scanning microscope (Olympus FV1000; Olympus, Hamburg, Germany) and 10× and 60× objectives. For each experiment, cells on two coverslips were used for each construct and 6–10 images were taken per condition. Z-stacks comprising the whole cells were additionally taken to confirm the surface localization of AC7. Image analysis was carried out using the ImageJ software (ImageJ version 1.53; https://imagej.nih.gov/ij/index.html; RRID: SCR_003070) and the JACoP plugin was used to calculate the Manders´ correlation values for intensities above Costes threshold and to calculate the Pearson´s correlation coefficient (Bolte and Cordelieres 2006).

## Statistics

GraphPad Prism 9 software (RRID: SCR_000306; GraphPad Software, Boston, MA, USA) was used to conduct the statistical analyses. Statistical tests used for comparisons are specified in the figure legends. With the Shapiro–Wilk test, normal distribution of the data was determined. For data with normal distribution, Student’s t test was used to compare two groups. Groups that were not normally distributed data were analyzed using one-way ANOVA with Bonferroni’s or Dunnett´s multiple comparison test. All numerical data are presented as group mean values with standard deviation (SD).

## Results

### Comparable Protein Expression

**Fig. 2:**
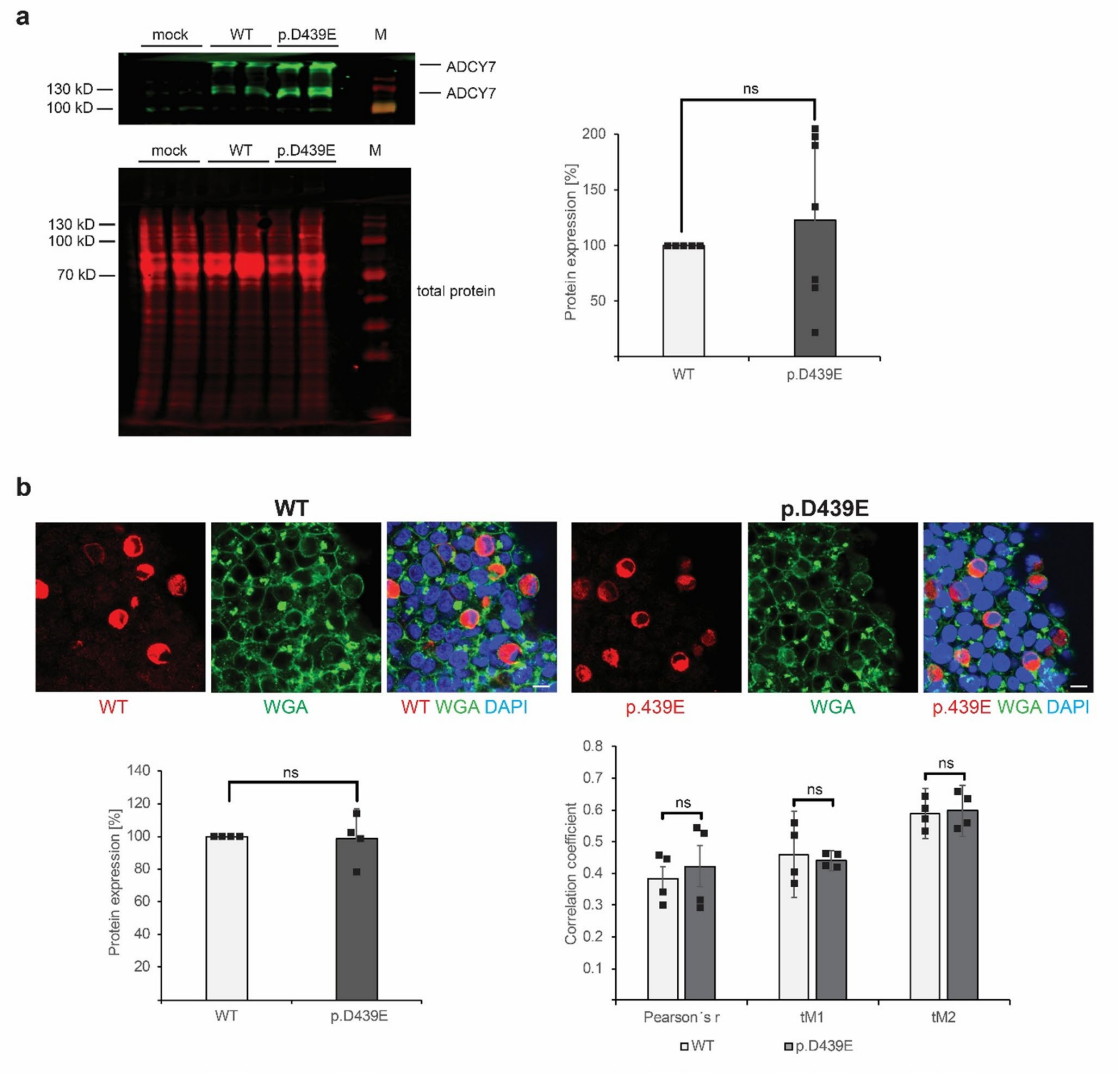
Unchanged expression of AC7 p.Asp439Glu (p.D439E). HEK293 cells were transfected with plasmids encoding WT AC7, p.Asp439Glu AC7 or with an empty vector and expression of AC7 was determined. (**a**) Cells were lysed and cell lysates subjected to Western blot analysis. Representative blots show AC7 and total protein staining which was used for normalization. The graph depicts the quantification of AC7 levels. M = molecular weight marker. (**b**) Immunofluorescence images show AC7 (red), WGA (green) and nuclei (blue). Scale bars: 20 μm. Graphs depict the quantification of total fluorescence (left graph) and the correlation coefficients (right graph). The Pearson’s correlation coefficient and the Manders’ overlap coefficients are shown. tM1: proportion of AC7 (red) overlapping with WGA (green) over the total intensity; tM2: proportion of WGA (green) overlapping with AC7 (red) over the total intensity. *N* = 5 for Western blots and *n* = 4 for immunostaining. Mean values and single values ± SD are shown. Data were analyzed with Student´s t test, *p* > 0.05, no significant differences (ns)

For comparison of the protein expression and localization of AC7 wildtype (WT) and the p.Asp439Glu variant, immunocytochemistry and Western blot analysis were performed. HEK293 cells were transfected with human *ADCY7* cDNA, lysed and used for Western blotting or fixed and used for immunostaining. Western blots of AC7 from lysates of WT and mutant-transfected cells were analyzed by densitometry and showed comparable expression levels between the two AC7 forms (*p* > 0.05, *n* = 5), suggesting that the p.Asp439Glu substitution does not affect protein abundance in HEK293 cells (Fig. 2a). In addition to analysis of total protein levels after cell lysis, immunostaining of HEK cells with an antibody detecting AC7 and with WGA to visualize the plasma membrane also allowed to determine if the AC7 p.Asp439Glu variant is also present at the membrane (Fig. 2b). The immunostaining confirmed the Western blot result of comparable expression of p.Asp439Glu and WT AC7 and similar levels of p.Asp439Glu and WT AC7 were observed at the plasma membrane (*p* > 0.05, *n* = 4; Fig. 2c). Results indicate that localization and expression of AC7 in HEK293 cells are not affected by the p.Asp439Glu exchange. It is noteworthy to mention, that WT and variant AC7 expression was not only observed at the plasma membrane but also in the cytosol (Fig. 2b). This staining might be due to internalized proteins or to proteins that are trafficking to the cell surface.

### Reduced Basal Activity of the p.Asp439Glu Variant

**Fig. 3:**
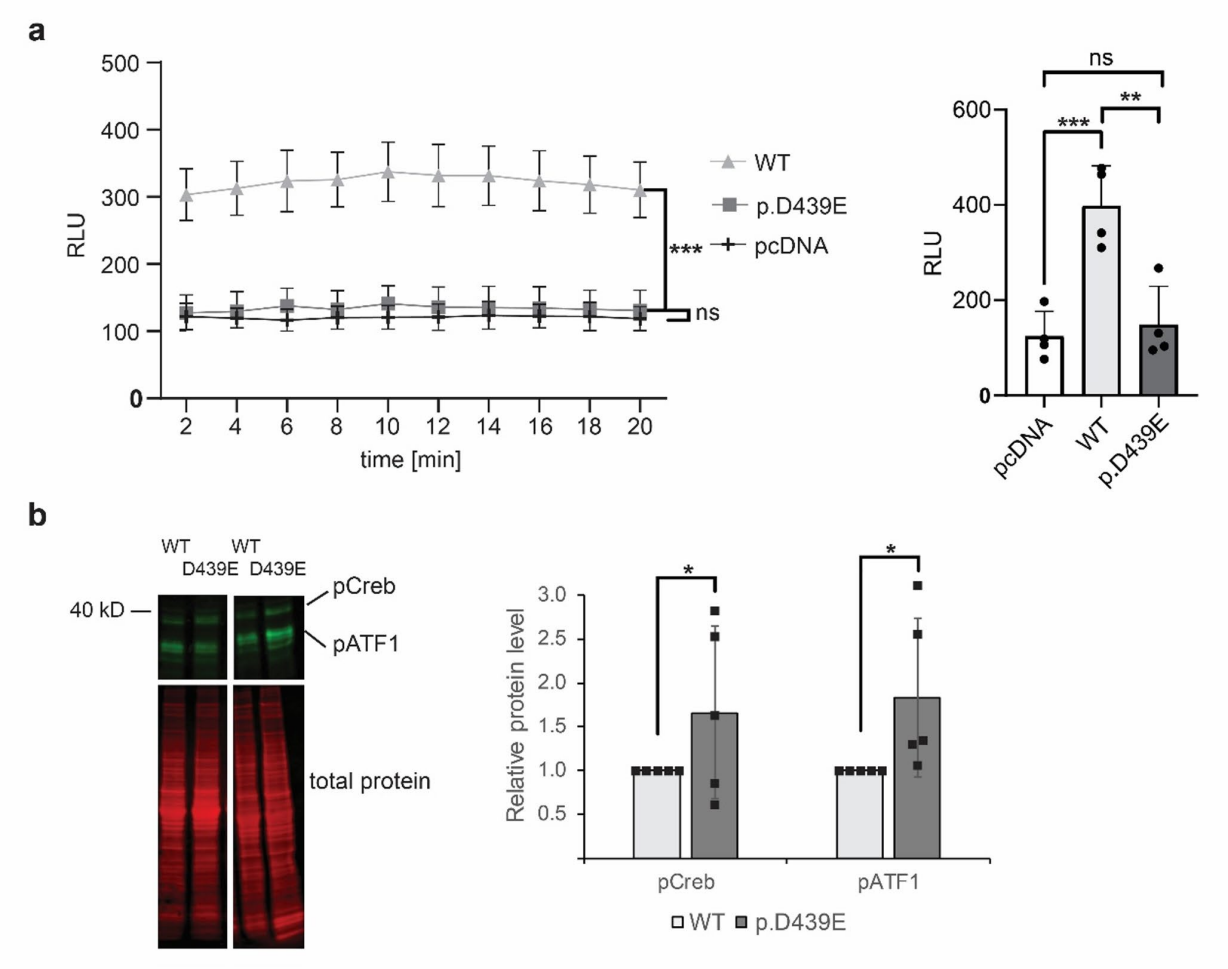
Reduced basal activity of p.Asp439Glu (p.D439E) and enhanced CREB phosphorylation. (**a**) cAMP levels in pcDNA mock-transfected HEK293 cells and cells expressing WT AC7 or p.Asp439Glu AC7 were monitored under basal conditions for 20 min and relative cAMP levels are determined. The left graph shows a representative experiment and the graph on the right shows the average activity obtained from four experiments. (**b**) Representative Western bot images and quantification pCREB and pATF1 levels in cell lysates from cells expressing WT AC7 or p.Asp439Glu AC7 at basal conditions. Bands from the same blot but not adjacent from each other are shown separated. *N* = 4 for cAMP measurements and *n* = 5 for Western blots. Mean values (all graphs) and single values (bar graphs) ± SD are shown. Data were analyzed with one-way ANOVA with Bonferroni’s multiple comparison test (Western blots) or Dunnett´s multiple comparison test (cAMP measurement), *p < 0.05, ** *p* < 0.01, *** *p* < 0.001, *p* > 0.05 no significant differences (ns)

Next, enzymatic activity of the p.Asp439Glu variant was investigated. Live-cell cAMP measurement in HEK cells expressing WT or p.Asp439Glu AC7 revealed that cells expressing the p.Asp439Glu variant had significantly reduced basal cAMP levels, amounting to ~ 35% of the signal observed in WT-transfected cells (*p* < 0.001; *n* = 4; Fig. 3a). Furthermore, the cAMP levels in p.Asp439Glu AC7 expressing cells did not differ from those of HEK cells that were transfected with the empty vector (pcDNA) and which only express endogenous ACs (*p* > 0.05; *n* = 4; Fig. 3a). This indicates a loss-of-function effect of the constitutive activity, which is consistent with previous reports, but obtained here in a dynamic, time-resolved cellular system.

### Enhanced Downstream Phosphorylation of CREB and ATF1

To investigate how the p.Asp439Glu variant affects down-stream signaling pathways, phosphorylation of CREB and ATF1 were assessed. Physiologically, adenylate cyclase activation increases phosphorylation of CREB and ATF1, which then bind to the DNA and regulate transcription (e.g. Tomczak et al.^ 2025^; Pizzoni et al. 2024). Due to the lower activity of the p.Asp439Glu variant, we hypothesized reduced phosphorylation of CREB and ATF1 in cells expressing the variant. In contrast, despite reduced basal cAMP levels, phosphorylation levels of CREB and ATF1 were significantly higher under resting conditions in p.Asp439Glu expressing cells compared to cells expressing WT AC7 (Fig. 3b). This suggests that endogenous AC isoforms or feedback mechanisms may maintain downstream signaling in HEK293 cells expressing p.Asp439Glu or that the variant activates CREB and ATF1 independently of the cAMP pathway (Sun et al. 1994).

### Impaired Responsiveness to PMA Stimulation

**Fig. 4:**
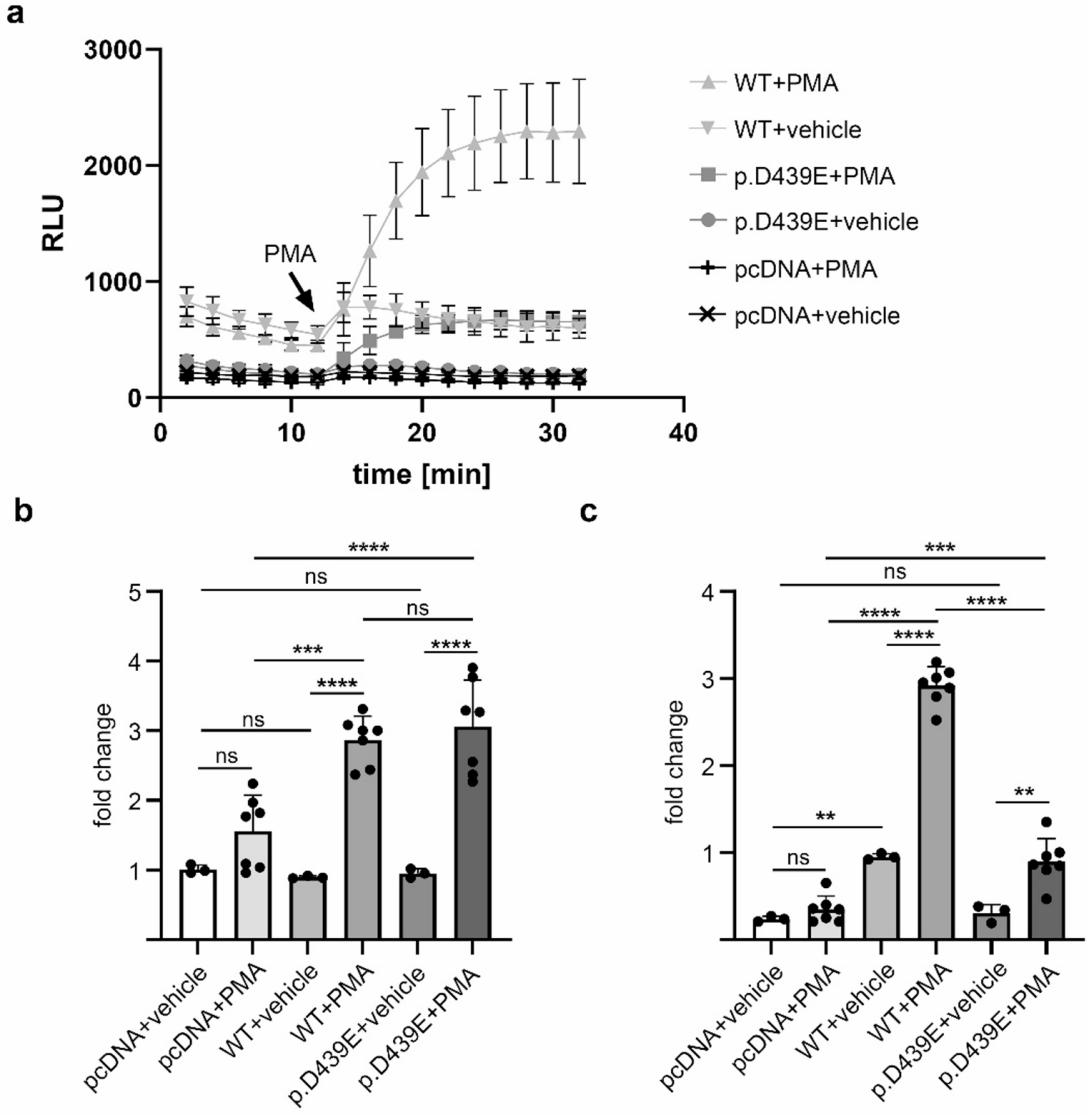
Impaired response to PKC stimulation of AC7 p.Asp439Glu (p.D439E). HEK293 cells were transfected with pcDNA or plasmids encoding WT AC7 and p.Asp439Glu AC7 and treated with vehicle solution or PMA to stimulate PKC. (**a**-**c**) cAMP levels in HEK293 were monitored under basal conditions and after stimulation with 1 µM PMA. Relative cAMP values from one representative experiment are shown (**a**). (**b**, **c**) Fold-changes after stimulation with PMA normalized to the corresponding baseline (**b**) or to the cAMP levels of WT AC7 expressing cells at basal conditions (**c**) are shown; *n* = 3 (stimulation with vehicle) and *n* = 7 stimulation with PMA). Mean values (all graphs) and single values (bar graphs) with SD are shown. Data were analyzed with one-way ANOVA with Tukey’s multiple comparisons test, *p < 0.05, **p < 0.01, ***p < 0.001, **** *p* < 0.0001, p > 0.05 no significant differences (ns)

Next, we tested whether PMA stimulation, which induces endogenous S1P generation, activates PKC and regulates cAMP production, could modulate AC7 activity (Brown et al. 2021). Upon PMA treatment (1 µM) cells expressing WT AC7 exhibited a pronounced increase in luminescence, indicating a robust cAMP response, whereas cells expressing the p.Asp439Glu variant showed a markedly attenuated response (Fig. 4a-c). Cells transfected with the pcDNA control displayed only a minimal response (Fig. 4a-c). Although the fold increase over baseline was similar in cells expressing p.Asp439Glu AC7 than in WT AC7–expressing cells (Fig. 4b), absolute cAMP levels remained substantially lower in the variant (Fig. 4a and c). These results indicate that the p.Asp439Glu AC7 variant retains responsiveness to PKC activation but is less efficient than WT AC7 in generating cAMP.

### Altered Responsiveness to S1P Stimulation

**Fig. 5:**
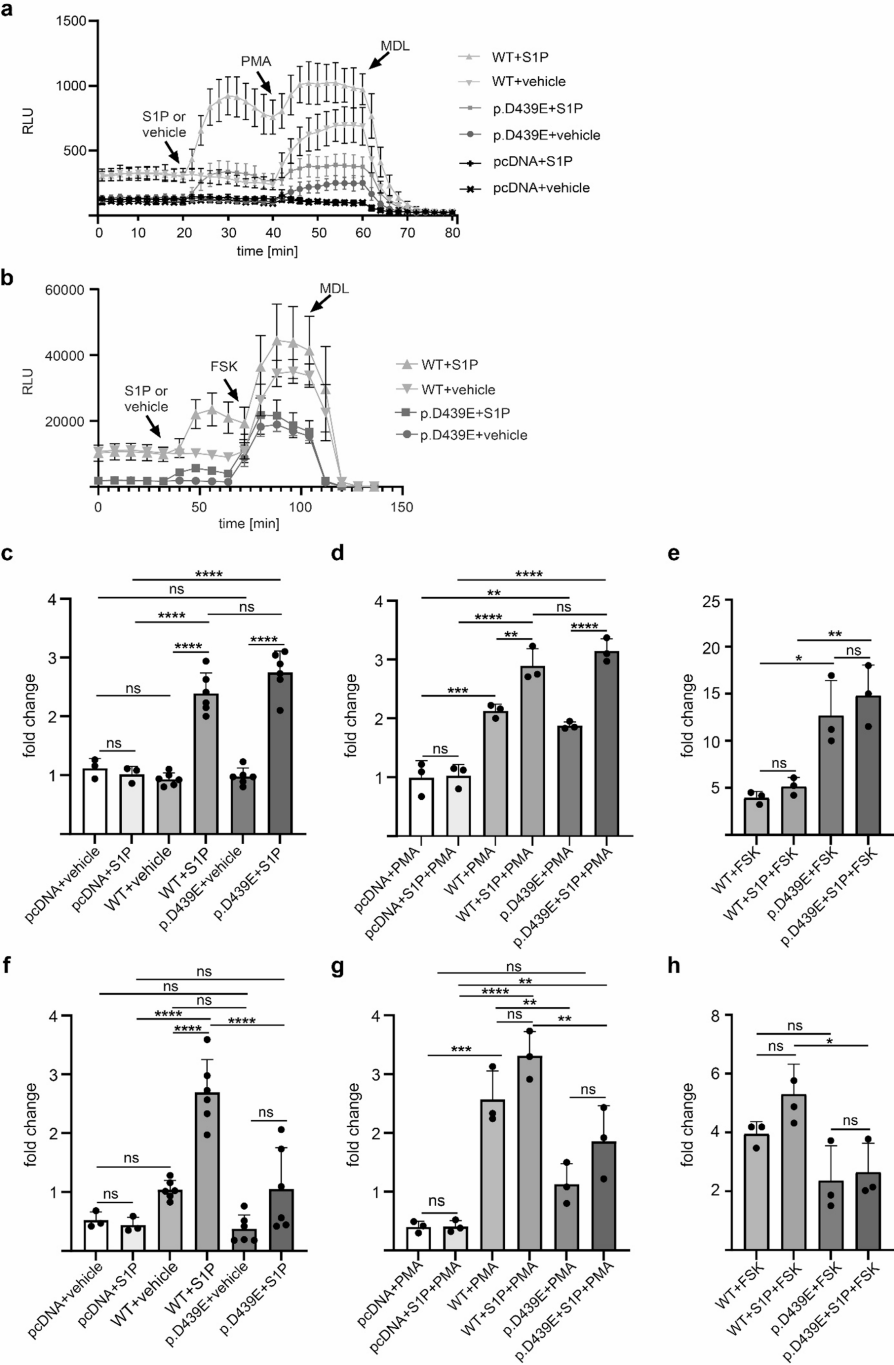
The AC7 p.Asp439Glu variant (p.D439E) shows an altered response to GPCR activation. HEK293 cells were transfected with pcDNA control or plasmids encoding WT AC7, p.Asp439Glu AC7. Cells were treated with vehicle solution or S1P to stimulate GPCRs followed by treatment with PMA to stimulate PKC or treatment with the AC7 activator forskolin (FSK) and finally the AC inhibitor MDL-12,330A (MDL) was applied. (**a**, **b**) cAMP levels in HEK293 cells were monitored under basal conditions, after vehicle or S1P stimulation, followed by PMA stimulation (**a**) or FSK stimulation (**b**) and MDL inhibition. Results from a representative experiment out of three experiments are shown. (**c**, **f**) Fold-changes in activity after S1P treatment when compared to the respective baseline (**c**) or compared to vehicle-treated WT (**f**) are shown. (**d**, **g**) Fold-changes in activity after S1P and PMA co-stimulation when compared to the respective baseline (**d**) or compared to vehicle-treated WT (**g**) are shown. (**e**, **h**) Fold-changes in activity after S1P and FSK co-stimulation when compared to the respective baseline (**e**) or compared to vehicle-treated WT (**h**) are shown. (**c**-**h**) *N* = 3 (pcDNA and WT AC7 and p.D439E AC7 stimulation with S1P and PMA or FSK), *n* = 6 (WT AC7 and p.D439E AC7, stimulation with vehicle or S1P); mean values and single values with SD are shown. Data were analyzed with one-way ANOVA with Tukey’s multiple comparisons test, * *p* < 0.05, **p < 0.01, ***p < 0.001, ****p < 0.0001, *p* > 0.05 no significant differences (ns)

To assess the response of p.Asp439Glu AC7 to S1P stimulation and GPCR activation and to determine if co-stimulation with S1P and PMA or FSK has synergistic effects on AC7 activity, HEK cells were treated with S1P or vehicle solution, followed by PMA and FSK treatment (Fig. 5a-h). S1P treatment led to enhanced cAMP levels in WT AC7- and p.Asp439Glu AC7-expressing cells (Fig. 5a, b, c) although absolute values in p.Asp439Glu AC7-expressing cells were significantly lower (Fig. 5a, b, f). Control cells not expressing AC7 had low endogenous cAMP levels and these levels remained much lower in these cells after application of S1P compared to levels in AC7-expressing cells (Fig. 5a-h). Since S1P treatment did not increase cAMP levels in control cells, we can exclude that the observed cAMP elevation in cells expressing WT or p.Asp439Glu AC7 is driven by activation of endogenous adenylate cyclases (Fig. 5c, f). Together with the finding that stimulated cAMP responses are markedly higher in AC7-expressing cells than in control cells, this verifies that the observed cAMP production after S1P stimulation is predominantly attributable to the transfected AC7 rather than to endogenous AC activity.

Interestingly, treatment with PMA after S1P stimulation did not lead to a further enhancement in cAMP levels in cells expressing WT and p.Asp439Glu AC7, while subsequent treatment with the AC7 activator FSK further enhanced cAMP levels in AC7 WT- and p.Asp439Glu-expressing cells (Fig. 5a, b,d, e,g, h). The adenylate cyclase inhibitor MDL-12,330 A strongly reduced the cAMP levels in all cells and values dropped below baseline levels 20 min after inhibitor treatment (Fig. 5a, b). When cAMP values were normalized to the mean of WT baseline, S1P, FSK and PMA treatment induced a higher response in AC7 WT expressing cells compared to p.Asp439Glu expressing cells (Fig. 5f-h).

Overall, these results indicate that the p.Asp439Glu variant retains some responsiveness to S1P but exhibits substantially reduced cAMP production compared to WT AC7, confirming a partial loss-of-function phenotype.

**Fig. 6:**
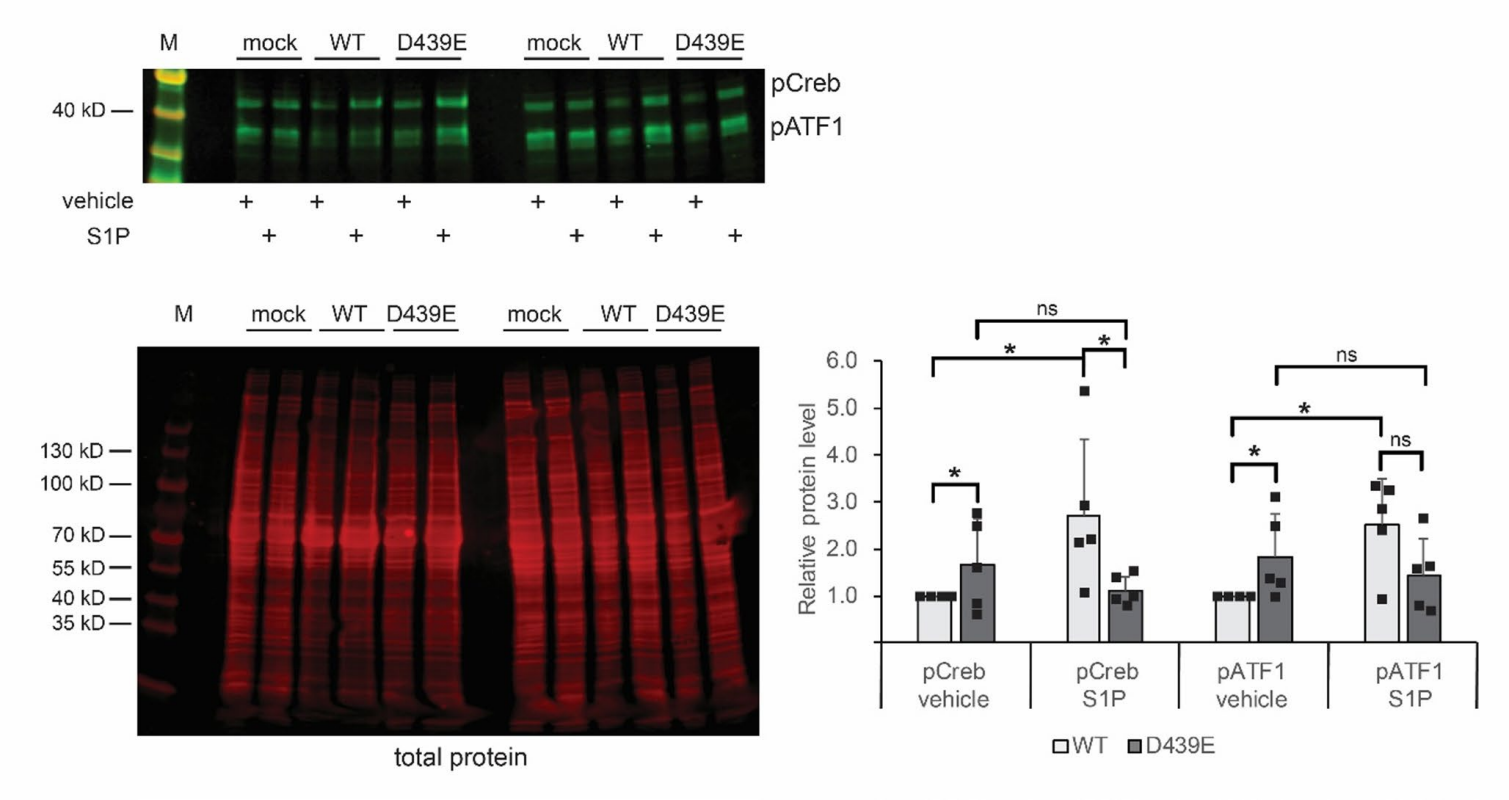
Altered down-stream signaling of p.Asp439Glu AC7. HEK293 cells were transfected with plasmids encoding WT AC7 or p.Asp439Glu AC7 and treated with vehicle solution or S1P to stimulate GPCRs. Western blot images show pCREB and pATF1 levels in cells with vehicle or S1P stimulation (top image, green bands) and total protein stain (lower image, red bands) used for normalization. M = molecular weight marker. The graph depicts the quantification of pCREB and pATF1 levels; *n* = 5. Mean values and single values with SD are shown. Data were analyzed with one-way ANOVA with Bonferroni’s multiple comparison test, p > 0.05 no significant differences (ns)

To further substantiate the notion that S1P stimulation differently affects WT AC7 and p.Asp439Glu AC7, phosphorylation of down-stream effectors of cAMP signaling was determined. Western blot analysis revealed that S1P stimulation increased phosphorylation of CREB and ATF1 in cells expressing WT AC7, whereas no stimulus-dependent increase was observed in cells expressing the p.Asp439Glu variant (Fig. 6). Notably, basal levels of phosphorylated CREB and ATF1 were elevated in cells expressing p.Asp439Glu AC7, suggesting aberrant baseline activation of these transcription factors. Together, these findings indicate that although the variant may retain some responsiveness to upstream GPCR signaling, its capacity to generate cAMP and to activate downstream effectors in a regulated, stimulus-dependent manner is impaired compared with WT AC7, resulting in altered downstream signaling.

## Discussion

*ADCY7* is a gene related to inflammation and diseases involving the immune system (Cardinale et al. 2025; Liu et al. 2025; Reeve et al. 2024; Shao et al. 2025). AC7 is related to rheumatoid arthritis susceptibility and suggested to modulate neutrophil and macrophage functions (Shao et al. 2025). Enhanced AC7 basal activity is found in UC patients (Rachmilewitz et al. 1983) and genome-wide association analyses revealed that the most risk-conferring genetic polymorphism for UC is the amino acid substitution p.Asp439Glu in the *ADCY7* gene (Luo et al. 2017). The AC7 p.Asp439Glu variant exhibits impaired basal catalytic activity and reduced AC7 function is linked to altered transcription in CD4^+^ T cells, including upregulation of Th2 cytokines (IL-4, IL-5, IL-13) and MHC class II molecules - patterns associated with UC pathology (Cardinale et al. 2025). Furthermore, the NLR family pyrin domain containing 3 (NLRP3) inflammasome plays a crucial role in the pathogenesis of UC (Ali et al. 2023; Zhen and Zhang 2019) and AC7 is required for NLRP3 inflammasome activation downstream of toll-like receptor 9 (TLR9) by catalyzing the generation of the dimeric form of cAMP (Liu et al. 2025). Thus, elucidating the functional consequences of amino acid substitutions in AC7 may enable the development of targeted AC7-based therapies to modulate inflammatory responses in ulcerative colitis.

According to published data, we postulated that the AC7 p.Asp439Glu variant might (i) be altered in membrane expression, (ii) exhibit impaired basal catalytic activity and show reduced downstream signaling. In our study in HEK293 cells, we found that the expression of the p.Asp439Glu variant was normal and that the membrane localization was retained, although both WT and p.Asp439Glu AC7 were also observed in the cytoplasm. However, basal cAMP generation of p.Asp439Glu was reduced and a markedly diminished response to PMA and S1P stimulation was observed indicating a strongly reduced response to GPCR and PKC activation, which confirms our initial hypothesis and previous studies. Interestingly, PMA and S1P stimulation of p.Asp439Glu AC7 resulted in similar fold-change activity compared with WT AC7, although absolute cAMP levels remained significantly lower. Co-stimulation with FSK led to a modest increase in cAMP levels in both WT and p.Asp439Glu AC7-expressing cells, whereas PMA co-stimulation did not further enhance cAMP production. One limitation of our study is that endogenous adenylate cyclases contribute to basal cAMP levels and to the increase observed after FSK stimulation. However, cells expressing WT AC7 reached substantially higher cAMP levels than cells expressing the p.Asp439Glu variant or pcDNA alone, indicating that a significant portion of the response is driven by the transfected WT AC7, as otherwise all conditions would be expected to reach similar levels.

S1P usually decreases cAMP levels in HEK293 cells, since the main S1P receptors are G_i_ coupled and inhibit ACs (Cheng et al. 2022; Drexler et al. 2021; Windh et al. 1999). In contrast to S1P receptor 1, which couples exclusively to G_i_ proteins, S1P receptors 2 and 3 couple to the G_i_, G_q_, and G_12/13_ families of heterotrimeric G-proteins (Means et al. 2008; Windh et al. 1999). In macrophages and other hematopoietic cell lineages, S1P acts through S1P receptor 2 and G_13_ and strongly enhances the cAMP rise triggered by G_s_ coupled receptors (Jiang et al. 2007, 2013). In these cells regulation from the G_13_ pathway requires the expression of AC7 (Jiang et al. 2008). HEK cells endogenously express S1P receptor 2 and 3 which can couple to G_i_, G_q_, and G_12/13_ (Drexler et al. 2021). S1P receptor activation leads to G_i_ dependent inhibition of AC3 and 6 endogenously expressed in these cells. In AC7 expressing HEK293 cells, S1P and G_s_ stimulation can recapitulate the G_13_ dependent enhancement of cAMP seen in macrophages (Jiang et al. 2008), showing that S1P treatment can activate AC7 and enhance cAMP levels in HEK cells. Thus, we suggest that S1P–G_12/13_ signaling increases cAMP by boosting of G_s_ stimulated AC7 activity in our experiments.

Although basal activity of p.Asp439Glu AC7 was markedly lower compared to the activity of WT AC7, phosphorylation levels of CREB and ATF1 were unexpectedly higher in cells expressing the p.Asp439Glu variant. GPCR stimulation only enhanced levels of phosphorylated CREB and ATF1 in WT AC7 expressing cells but not in the AC7 variant expressing cells. At basal conditions, endogenous ACs, phosphodiesterases, EPACs, enhanced GPCR activity or higher activity of protein kinase A or MAP kinases might compensate for the p.Asp439Glu AC7 loss-of-function and contribute to the enhanced phosphorylation of CREB and ATF1. Since only cells expressing WT AC7 exhibited enhanced phosphorylation of CREB and ATF1 after GPCR activation, we assume that phosphorylation of CREB and ATF1 is mainly regulated by the overexpressed WT AC7 and hypothesize that p.Asp439Glu loss-of-function is not compensated by endogenous ACs when GPCRs are activated. Although the variant still responds to S1P stimulation to some extent, the subtle changes in cAMP levels do not lead to detectable changes in CREB and ATF1 phosphorylation using our Western blot–based quantification approach. We suggest that expression of the p.Asp439Glu variant could lead to alterations in gene expression or localization and activity of other proteins involved in GPCR signaling. This new hypothesis needs to be investigated in further studies.

Together, our findings suggest that the ulcerative colitis-associated p.Asp439Glu AC7 variant disrupts GPCR signaling and that activation of the downstream signaling cascade via AC7 is dysfunctional in the variant, leading to a loss of regulated, stimulus-induced cAMP signaling even if compensatory basal phosphorylation of CREB/ATF1 persists. The assumption of an impaired down-stream signaling of p.Asp439Glu expressing cells leading to altered gene transcription, is in line with the current finding of differentially regulated genes in transcriptome analysis of *ADCY7* knock-down human primary CD4^+^ T cells (Cardinale et al. 2025). Since we found normal p.Asp439Glu protein expression, we can attribute the reduced cAMP generation to impaired enzyme activity while previously this distinction was not possible because lower expression and lower activity of the p.Asp439Glu protein were observed. Our results in HEK293 cells provide biochemical validation for reduced activity of the p.Asp439Glu variant and extend the understanding of signal transduction integrity under variant conditions.

Of note, AC7 is not only present at the membrane but it undergoes subcellular translocation via caveolae-mediated endocytosis and then translocates to the nucleus with the help of leucine-rich repeat-containing protein 59 and karyopherin subunit beta. In the nucleus, it functions as a transcription cofactor of CCAAT/enhancer binding protein alpha to induce chemokine (C-C motif) ligand 5 (*CCL5)* transcription, thereby increasing CD8^+^ T cell infiltration to restrain hepatocellular carcinoma progression. Furthermore, AC7 can be secreted as exosomes and enter neighboring tumor cells to promote CCL5 induction (Chen et al. 2024). Thus, it is unlikely that the intracellular proteins observed in our study are an overexpression artifact or mislocalized proteins. It would be of interest to investigate the endocytosis and nuclear translocation as well as the function as transcriptional cofactor and the promotor binding of the AC7 variants to better understand the effect of amino acid changes in the AC7 protein.

Of note, our and previous studies show that dysregulated activity and expression of p.Asp439Glu AC7 lead to an imbalance in cAMP generation and GPCR signaling pathways (Cardinale et al. 2025). cAMP signaling is subject to tight and dynamic regulation under both physiological and pathophysiological conditions. GPCR-dependent regulation of cAMP together with absolute basal levels is essential for immune and epithelial cell signaling during UC progression and is also central to therapeutic mechanisms. If AC7 is unable to respond appropriately to GPCR stimulation, as we show for the variant, then crucial regulatory pathways become dysfunctional. This impaired signal responsiveness provides a plausible mechanistic explanation for why a variant with reduced modulatory capacity can still confer increased UC risk.

From a therapeutic standpoint, the partial AC7 activity can probably not be enhanced to normal levels by treatment with GPCR agonists like S1P or agents acting directly on AC7. Thus, the potential efficacy of S1P receptor agonists, such as ozanimod and etrasimod, in patients harboring this variant should be investigated in future studies. If indeed carriers of the p.Asp439Glu and possibly other functionally impaired variants should respond differently to S1P receptor modulators or cAMP-elevating drugs, this may pave the way for stratified or precision therapy in UC.

Limitations of our study include the use of HEK293 cells, which do not fully recapitulate the immune environment. Although this model allows controlled biochemical analysis, future studies should assess the p.Asp439Glu variant in primary human immune cells or organoid cultures to evaluate context-dependent responses.

## Conclusion

The UC-associated AC7 p.Asp439Glu variant exhibits markedly reduced basal cAMP levels and profoundly impaired PMA- and S1P-induced signaling in live-cell assays, consistent with a partial loss-of-function effect. Given the immune-restricted expression of AC7, these findings underscore the need to explore cAMP-modulating strategies in precision approaches for ulcerative colitis. Rather than broadly targeting GPCR or cAMP signaling, isoform-specific modulation of adenylyl cyclases or phosphodiesterases may offer therapeutic benefit with fewer off-target effects, particularly where AC7-dependent GPCR signaling is compromised.

## Data Availability

The datasets generated and analyzed during the current study are available from the corresponding author on reasonable request.

## References

[CR1] Ali FEM, Ibrahim IM, Ghogar OM, Abd-Alhameed EK, Althagafy HS, Hassanein EHM (2023) Therapeutic interventions target the NLRP3 inflammasome in ulcerative colitis: Comprehensive study. World J Gastroenterol 29:1026–1053. 10.3748/wjg.v29.i6.1026 36844140 10.3748/wjg.v29.i6.1026 PMC9950862

[CR2] Binkowski BF, Butler BL, Stecha PF, Eggers CT, Otto P, Zimmerman K, Vidugiris G, Wood MG, Encell LP, Fan F, Wood KV (2011) A luminescent biosensor with increased dynamic range for intracellular cAMP. ACS Chem Biol 6:1193–1197. 10.1021/cb200248h 21932825 10.1021/cb200248h

[CR3] Bolte S, Cordelieres FP (2006) A guided tour into subcellular colocalization analysis in light microscopy. J Microsc 224:213–232. 10.1111/j.1365-2818.2006.01706.x 17210054 10.1111/j.1365-2818.2006.01706.x

[CR4] Brown RDR, Veerman BEP, Oh J, Tate RJ, Torta F, Cunningham MR, Adams DR, Pyne S, Pyne NJ (2021) A new model for regulation of sphingosine kinase 1 translocation to the plasma membrane in breast cancer cells. J Biol Chem 296:100674. 10.1016/j.jbc.2021.100674 33865856 10.1016/j.jbc.2021.100674 PMC8135045

[CR5] Cardinale CJ, Liu Y, Kevadia A, Strong A, Watts VJ, Hakonarson H (2025) The ulcerative colitis risk gene adenylyl cyclase 7 restrains the T-helper 2 phenotype and Class II antigen presentation. J Crohns Colitis 19:jjaf030. 10.1093/ecco-jcc/jjaf030 39957491 10.1093/ecco-jcc/jjaf030 PMC11920793

[CR6] Chen J, Jiang Y, Hou M, Liu C, Liu E, Zong Y, Wang X, Meng Z, Gu M, Su Y, Wang H, Fu J (2024) Nuclear translocation of plasma membrane protein ADCY7 potentiates T cell-mediated antitumour immunity in HCC. Gut 74:128–140. 10.1136/gutjnl-2024-332902 39349007 10.1136/gutjnl-2024-332902 PMC11671903

[CR7] Cheng L, Su L, Tian X, Xia F, Zhao C, Yan W, Shao Z (2022) A Pipeline to Investigate the Structures and Signaling Pathways of Sphingosine 1-Phosphate Receptors. J Vis Exp doi. 10.3791/64054 10.3791/64054 35758708

[CR8] Conley JM, Brand CS, Bogard AS, Pratt EP, Xu R, Hockerman GH, Ostrom RS, Dessauer CW, Watts VJ (2013) Development of a high-throughput screening paradigm for the discovery of small-molecule modulators of adenylyl cyclase: identification of an adenylyl cyclase 2 inhibitor. J Pharmacol Exp Ther 347:276–287. 10.1124/jpet.113.207449 24008337 10.1124/jpet.113.207449 PMC3807067

[CR9] Devasani K, Yao Y (2022) Expression and functions of adenylyl cyclases in the CNS. Fluids Barriers CNS 19:23. 10.1186/s12987-022-00322-2 35307032 10.1186/s12987-022-00322-2 PMC8935726

[CR10] Drexler Y, Molina J, Mitrofanova A, Fornoni A, Merscher S (2021) Sphingosine-1-Phosphate Metabolism and Signaling in Kidney Diseases. J Am Soc Nephrol 32:9–31. 10.1681/ASN.2020050697 33376112 10.1681/ASN.2020050697 PMC7894665

[CR11] Eden N, Gaunt E, Ong EMS, Sharif K, Selinger C (2025) The Role of Novel Small Molecule Drugs in the Management of Inflammatory Bowel Disease. Br J Hosp Med (Lond) 86:1–14. 10.12968/hmed.2024.0798 40554447 10.12968/hmed.2024.0798

[CR12] Gao Y, Luo Y, Ji G, Wu T (2024) Functional and pathological roles of adenylyl cyclases in various diseases. Int J Biol Macromol 281:136198. 10.1016/j.ijbiomac.2024.136198 39366614 10.1016/j.ijbiomac.2024.136198

[CR13] Guo R, Liu T, Shasaltaneh MD, Wang X, Imani S, Wen Q (2022) Targeting Adenylate Cyclase Family: New Concept of Targeted Cancer Therapy. Front Oncol 12:829212. 10.3389/fonc.2022.829212 35832555 10.3389/fonc.2022.829212 PMC9271773

[CR15] Jiang LI, Collins J, Davis R, Lin KM, DeCamp D, Roach T, Hsueh R, Rebres RA, Ross EM, Taussig R, Fraser I, Sternweis PC (2007) Use of a cAMP BRET sensor to characterize a novel regulation of cAMP by the sphingosine 1-phosphate/G13 pathway. J Biol Chem 282:10576–10584. 10.1074/jbc.M609695200 17283075 10.1074/jbc.M609695200 PMC2526465

[CR14] Jiang LI, Collins J, Davis R, Fraser ID, Sternweis PC (2008) Regulation of cAMP responses by the G12/13 pathway converges on adenylyl cyclase VII. J Biol Chem 283:23429–23439. 10.1074/jbc.M803281200 18541530 10.1074/jbc.M803281200 PMC2516994

[CR16] Jiang LI, Wang JE, Sternweis PC (2013) Regions on adenylyl cyclase VII required for selective regulation by the G13 pathway. Mol Pharmacol 83:587–593. 10.1124/mol.112.082446 23229509 10.1124/mol.112.082446 PMC3583495

[CR17] Liu Q, Tang Z, Qian Y, Wang C, Kong C, Li M, Geng X, Zhang Y, Cheng X, Ren C, Wang K, Bai L, Wang L, Jiang D, Wang S, Liu X, Xia P (2025) Eukaryotic ADCY7 catalyzes the production of c-di-AMP to activate the NLRP3 inflammasome. Nat Chem Biol 21:1283–1291. 10.1038/s41589-025-01919-y 40419769 10.1038/s41589-025-01919-y

[CR18] Luo Y, de Lange KM, Jostins L, Moutsianas L, Randall J, Kennedy NA, Lamb CA, McCarthy S, Ahmad T, Edwards C, Serra EG, Hart A, Hawkey C, Mansfield JC, Mowat C, Newman WG, Nichols S, Pollard M, Satsangi J, Simmons A, Tremelling M, Uhlig H, Wilson DC, Lee JC, Prescott NJ, Lees CW, Mathew CG, Parkes M, Barrett JC, Anderson CA (2017) Exploring the genetic architecture of inflammatory bowel disease by whole-genome sequencing identifies association at ADCY7. Nat Genet 49:186–192. 10.1038/ng.3761 28067910 10.1038/ng.3761 PMC5289625

[CR19] Means CK, Miyamoto S, Chun J, Brown JH (2008) S1P1 receptor localization confers selectivity for Gi-mediated cAMP and contractile responses. J Biol Chem 283:11954–11963. 10.1074/jbc.M707422200 18296752 10.1074/jbc.M707422200 PMC2335351

[CR20] Nelson EJ, Hellevuo K, Yoshimura M, Tabakoff B (2003) Ethanol-induced phosphorylation and potentiation of the activity of type 7 adenylyl cyclase. Involvement of protein kinase C delta. J Biol Chem 278:4552–4560. 10.1074/jbc.M210386200 12454008 10.1074/jbc.M210386200

[CR21] Ostrom KF, LaVigne JE, Brust TF, Seifert R, Dessauer CW, Watts VJ, Ostrom RS (2022) Physiological roles of mammalian transmembrane adenylyl cyclase isoforms. Physiol Rev 102:815–857. 10.1152/physrev.00013.2021 34698552 10.1152/physrev.00013.2021 PMC8759965

[CR34] Pizzoni A, Zhang X, Altschuler DL (2024) From membrane to nucleus: A three-wave hypothesis of cAMP signaling. J Biol Chem 300: 105497 10.1016/j.jbc.2023.105497 10.1016/j.jbc.2023.105497 PMC10788541 38016514

[CR22] Rachmilewitz D, Karmeli F, Selinger Z (1983) Increased colonic adenylate cyclase activity in active ulcerative colitis. Gastroenterology 85:12–16 6303887

[CR23] Raker VK, Becker C, Steinbrink K (2016) The cAMP Pathway as Therapeutic Target in Autoimmune and Inflammatory Diseases. Front Immunol 7:123. 10.3389/fimmu.2016.00123 27065076 10.3389/fimmu.2016.00123 PMC4814577

[CR24] Reeve M, Kanai M, Graham D, Karjalainen J, Luo S, Kolosov N, Adams C, Ritari J, Karczewski K, Kiiskinen T, Fuller Z, Mehtonen J, Kurki M, Khan Z, Partanen J, McCarthy M, Artomov M, Tuomi T, Pirinen M, Kero J, Xavier R, Daly M, Ripatti S, Gen F (2024) Autoimmune hypothyroidism GWAS reveals independent autoimmune and thyroid-specific contributions and an inverse relation with cancer risk. Res Sq rs 3:rs–4626646. 10.21203/rs.3.rs-4626646/v1

[CR25] Serezani CH, Ballinger MN, Aronoff DM, Peters-Golden M (2008) Cyclic AMP: master regulator of innate immune cell function. Am J Respir Cell Mol Biol 39:127–132. 10.1165/rcmb.2008-0091TR 18323530 10.1165/rcmb.2008-0091TR PMC2720142

[CR26] Shao S, Zeng W, Zhang J, Ma L, Huang F, Jiang Z (2025) Decoding rheumatoid arthritis: Biomarker identification and immune profiling via bioinformatics and Mendelian randomization. Med (Baltim) 104:e43872. 10.1097/MD.0000000000043872 10.1097/MD.0000000000043872 PMC12385112 40859487

[CR27] Sun P, Enslen H, Myung PS, Maurer RA (1994) Differential activation of CREB by Ca2+/calmodulin-dependent protein kinases type II and type IV involves phosphorylation of a site that negatively regulates activity. Genes Dev 8:2527–2539. 10.1101/gad.8.21.2527 7958915 10.1101/gad.8.21.2527

[CR28] Sutkeviciute I, Vilardaga JP (2020) Structural insights into emergent signaling modes of G protein-coupled receptors. J Biol Chem 295:11626–11642. 10.1074/jbc.REV120.009348 32571882 10.1074/jbc.REV120.009348 PMC7450137

[CR29] Tavares LP, Negreiros-Lima GL, Lima KM, PMR ES, Pinho V, Teixeira MM, Sousa LP (2020) Blame the signaling: Role of cAMP for the resolution of inflammation. Pharmacol Res 159:105030. 10.1016/j.phrs.2020.105030 32562817 10.1016/j.phrs.2020.105030

[CR33] Tomczak J, Kapsa A, Boczek T (2025) Adenylyl Cyclases as Therapeutic Targets in Neuroregeneration. Int J Mol Sci 26: 6081. 10.3390/ijms26136081 10.3390/ijms26136081 PMC12249641 40649859

[CR30] Windh RT, Lee MJ, Hla T, An S, Barr AJ, Manning DR (1999) Differential coupling of the sphingosine 1-phosphate receptors Edg-1, Edg-3, and H218/Edg-5 to the G(i), G(q), and G(12) families of heterotrimeric G proteins. J Biol Chem 274:27351–27358. 10.1074/jbc.274.39.27351 10488065 10.1074/jbc.274.39.27351

[CR31] Zhen Y, Zhang H (2019) NLRP3 Inflammasome and Inflammatory Bowel Disease. Front Immunol 10:276. 10.3389/fimmu.2019.00276 30873162 10.3389/fimmu.2019.00276 PMC6403142

[CR32] Zhou F, Tichy AM, Imambocus BN, Sakharwade S, Rodriguez Jimenez FJ, Gonzalez Martinez M, Jahan I, Habib M, Wilhelmy N, Burre V, Lomker T, Sauter K, Helfrich-Forster C, Pielage J, Grunwald Kadow IC, Janovjak H, Soba P (2023) Optimized design and in vivo application of optogenetically functionalized Drosophila dopamine receptors. Nat Commun 14:8434. 10.1038/s41467-023-43970-0 38114457 10.1038/s41467-023-43970-0 PMC10730509

